# Inbound and outbound medical travel in Austria

**DOI:** 10.1108/JHOM-04-2020-0129

**Published:** 2020-12-15

**Authors:** August Österle, Carina Diesenreiter, Barbara Glinsner, Eva Reichel

**Affiliations:** Department of Socioeconomics, Vienna University of Economics and Business , Vienna, Austria; Centre of Social Innovation , Vienna, Austria; Medical University of Vienna , Vienna, Austria

**Keywords:** Medical tourism, Cross-border health care, Patient mobility, Europe, Framework

## Abstract

**Purpose:**

The purpose of this paper is twofold: First, it analyzes demand and supply-side factors that influence patient flows to and from Austria. Second, building on the empirical research and existing conceptualizations, the study offers a general extended framework to guide future comparative analysis.

**Design/methodology/approach:**

The paper draws on multiple data sources including a literature review, secondary data, website analysis and semi-structured interviews with patients and health providers. Content analysis was carried out to identify common motives for seeking care abroad and providers' orientation towards medical travel.

**Findings:**

Outbound medical travel is largely determined by factors of access, affordability and vicinity, while inbound medical travel is predominately driven by a lack of adequate medical infrastructure in source countries and quality, both in terms of medical and service quality. Providers distinguish themselves according to the extent they take part in medical travel.

**Research limitations/implications:**

The findings emerging from a single country case study approach cannot be generalized across settings and contexts, albeit contributing to a better understanding of current medical travel patterns in Europe.

**Originality/value:**

Unlike most recent contributions, this study focuses both on inbound and outbound medical travel in Austria and investigates patient flows for distinctive treatments and drivers. While analysis of the supply-side of medical travel is often limited to tourism studies, this study provides a critical insight into developments in Europe from a health policy perspective, acknowledging that diverse medical travel patterns in Europe coexist.

## 1. Introduction

Traveling across borders for health care – or medical travel, as commonly termed – has grown considerably in the past decades ( Horsfall and Lunt, 2015b ). As a result, cross-border patient mobility has become a major issue in health care policies in Europe, not least in the context of European social security coordination ( Greer, 2014 ; Vollaard and Martinsen, 2017 ) and the general possibility to travel abroad as “patient-consumer” in search of care ( Ormond and Sothern, 2012 , p. 936). Beyond intra-EU developments, medical travel in Europe also involves the movement of EU citizens outside the EU for receiving care as well as the purposeful travel of non-EU patients into the EU. However, compared to the US experience of medical travel and efforts of Asian countries in meeting the growing demand for health-related travel ( Bochatan, 2015 ; Pocock and Phua, 2011 ; Whittaker and Chee, 2015 ; Medhekar *et al.* , 2019 ), medical travel in Europe is less researched ( Carrera and Lunt, 2010 ). At a European level, studies have focused primarily on specific health provisions, such as dental care ( Kovacs and Szocska, 2013 ; Österle *et al.* , 2009 ), or on particular cross-border situations and country case studies ( Levaggi and Montefiori, 2014 ; Dryglas and Lubowiecki-Vikuk, 2019 ), and more explicitly for the UK and its implications on the NHS (see, e.g. Hanefeld *et al.* , 2014 ; Lunt *et al.* , 2014b ; Lunt *et al.* , 2013 ; Pagán and Horsfall, 2019 ). A broader mapping of medical travel developments for other European countries, considering both inbound and outbound medical travel, and equally addressing demand and supply-side determinants, is still missing.

The aim of this article is to contribute to existing literature by providing first empirical insights into medical travel to and from Austria. Located in the center of Europe, Austria is a particularly interesting case, as it not only serves as popular source country of medical travelers, it also has a longstanding tradition in treating patients from abroad. Contrary to most recent literature, this study addresses both inbound and outbound patient flows in terms of procedures and drivers to explore the different dynamics and patterns that shape contemporary medical travel in Europe. More specifically, the paper investigates the main characteristics of patients entering or leaving Austria for medical reasons, it studies their motives for travelling cross-border, and it discusses the role of providers and intermediaries in facilitating medical travel. Hence, it includes both demand and supply-side considerations of currently observable patient flows. By capturing the nature of both inbound and outbound medical travel in Austria, the study disentangles medical travel patterns in the broader European region and classifies patient movements to and from Austria in existing conceptual frameworks, in particular those developed by Glinos *et al.* (2010) and Österle *et al.* (2013) . In a discussion, this analysis is then used to suggest an extended theoretical framework applicable for studying medical travel in health management and policy research. Existing frameworks have mainly emphasized patient motives and the legal context for accessing health care across borders. A broader coverage of the supply-side perspective is mostly limited to tourism and marketing studies. The framework proposed here will cover both demand and supply-side determinants and consider the perspectives of patients, providers, and intermediaries to guide further comparative analysis in this field.

In the next Section 2 , this paper provides a brief state-of-the-art overview to the conceptualization of medical travel. After outlining the materials and methods used in Section 3 , Section 4 explores inbound and outbound medical travel in Austria. Building on these findings and the existing literature, Section 5 highlights the implications of this study and suggests an extended conceptual framework. Section 6 then concludes with a summary of key aspects and unanswered questions that need to be addressed in future research.

## 2. Conceptualizations of medical travel

At present, medical travel is used as umbrella term for a highly diverse cross-border phenomenon, including travel for care between and within high, middle and low income countries ( Connell, 2013 , p. 5; Lunt *et al.* , 2013 , p. 2). Patients might travel long distances to obtain necessary treatments, go abroad to consume more affordable elective procedures, live in border-regions and receive care in a neighboring country, or seek treatments that are inaccessible or even illegal at home. Furthermore, medical travel comprises a wide range of available treatments, ranging from essential interventions to discretionary ones. The latter often include health provisions such as cosmetic surgery, ophthalmology, dental care, orthopedics, bariatrics and assisted reproductive technologies ( Lunt *et al.* , 2011 ; Glinos *et al.* , 2010 ). This variety of treatments and geographic locations as well as the present industrial desire to market medical travel have made attempts to define patient movements extremely difficult. Therefore, definitions of medical travel – or medical tourism, as these terms are often used interchangeably – tend to be broad and unspecified ( Connell, 2013 , 2015 ).

In this article, we draw on more precise definitions to differentiate medical travel from other forms of health-related travel. Carrera and Bridges (2006 , p. 447) describe medical tourism as “the organized travel outside one's natural healthcare jurisdiction for the enhancement or restoration of the individual's health through medical intervention.” According to this approach, medical travel has derived from a broader concept of health tourism and distinguishes itself due to three crucial factors: the geographical setting, the type of health intervention, and the structural facility where services are delivered. By drawing on this definition, we only consider planned medical interventions, both inpatient and outpatient, which are carried out in (private or public) foreign hospitals, specialized clinics or individual practices and thus exclude therapeutic and passive health services such as massages.

Similarly to Glinos *et al.* (2010 , p. 1,145), we aim at investigating “the movement of a patient travelling to another country to seek planned health care.” What follows from this definition is that medical travel, as we discuss it, differs from other forms of patient mobility, e.g. emergency care use by temporary visitors or patients sent abroad by their statutory health insurance ( Bertinato *et al.* , 2005 ). Medical travel implies patients' purposeful and deliberate travel outside the country of residence, where they may or may not be covered by national health insurance. In the institutional context of the EU and based on the principles of free movement, EU citizens can make use of transnational social security rights that allow them to access planned care in another EEA member state and, under certain requirements, get treatment costs reimbursed by their national health institution ( European Commission, 2019 ). As Carrera and Lunt (2010) point out in this context, European patients travelling for care thus may be conceptualized in dual roles of consumers and citizens, which again poses new terminological difficulties. With regard to the EU context, however, the terms cross-border health care and cross-border patient mobility appear to be more commonly used ( Legido-Quigley *et al.* , 2012 , p. 27; Turner and Hodges, 2012 , p. 7). Nevertheless, unlike Glinos *et al.* (2010) , we consciously choose the term medical travel instead of patient mobility, as our discussion involves both a demand-side approach on the individual motives for seeking care abroad and a supply-side perspective focusing on providers and intermediaries and their possible ambitions to facilitate medical travel.

Apart from the general awareness that medical travel can take multiple forms and has a longstanding tradition ( Smith and Puczkó, 2014 ; Lunt *et al.* , 2013 ), current research primarily focuses on medical travel from high to lower income countries. According to Lunt *et al.* (2011) , the 21st century form of “medical tourism” is characterized by large numbers of patients travelling, a reversed patient flow from more to less developed countries and an industry development facilitated by new infrastructure, including cheaper travel opportunities and easier information provision via Internet. Likewise, Connell (2013) states that a “reversed globalization” has occurred in the last 2 decades, meaning the travel for care to less industrialized countries driven by a combination of factors such as costs, access, service, and quality. Hence, contributions often concentrate on these *new* key features, especially the industrial desire of destinations to promote medical travel. At the same time, *older* forms of patient mobility, according to which people from countries with less advanced health care systems travel to nations with better facilities and specialized staff, remain largely unconsidered in recent debates, even though these might have also increased in the wake of globalization. So far only relatively few studies address this gap and focus on patient mobility from low and middle income countries to similar or higher income countries ( Durham and Blondell, 2017 ; Ormond and Sulianti, 2017 ; Snyder *et al.* , 2015 ).

In this paper, we consider both patient flows to and from Austria and provide evidence for a co-existence of these “old” and “new” medical travel patterns characterizing medical travel in Europe. We categorize examined empirical motives of inbound and outbound medical travel in Austria according to existing conceptual frameworks. In particular, we use the proposed typology of cross-border patient mobility by Glinos *et al.* (2010) and the framework of the demand for transnational medical travel developed by Österle *et al.* (2013) . The former typology classifies patient mobility by cross-referencing its motivations with the insurance status of a patient. The authors distinguish four reasons why patients obtain care abroad: availability, affordability, familiarity and perceived quality. According to the framework by Österle *et al.* (2013) , patients travel abroad for care that is more affordable, of better quality, or offers easier access compared to provision in their home countries. Other influential factors include cultural and social factors as well as the institutional environment.

Irrespective of the direction of patient flows, reasons why people decide to travel across borders for care are highly diverse and depend on individual circumstances as well as institutional settings. While existing typologies tend to focus on the demand for medical travel, this paper more closely links the demand-side perspective with that of suppliers and intermediaries and their attitudes towards medical travel. This will finally contribute to the development of an extended framework for studying medical travel, covering both demand and supply-side determinants and considering the diversity of medical travel patterns in Europe.

## 3. Materials and methods

The study draws on multiple data sources including a literature review, secondary data, Website searches, and interviews. It uses empirical findings from two projects, one focusing on incoming international patients receiving inpatient care in Austria and one studying outgoing Austrian patients in search for dental and ophthalmological procedures in the broader Central Eastern European region. In each project, a systematic analysis of websites of providers and intermediaries that promote medical travel was undertaken. Since the Internet is one of the key drivers of contemporary medical travel ( Horsfall *et al.* , 2013 ; Horsfall and Lunt, 2015a ), content analysis of websites has become an appropriate strategy for gathering information on medical travel developments. While published methodologies differ on how they are explicitly adopted, many studies have shown that a systematic review of medical travel websites contributes to a better understanding of medical travel, especially its industry development ( Cormany and Baloglu, 2011 ; Frederick and Gan, 2015 ; Moghavvemi *et al.* , 2017 ; Lunt and Carrera, 2011 ).

Besides research on Austrian patients travelling abroad for dental care, which has already been studied in more detail ( Kovacs and Szocska, 2013 ; Österle *et al.* , 2009 ), we include outbound travel for ophthalmological care in our analysis. Ophthalmological treatments, particularly refractive surgery to correct myopia (e.g. LASIK treatments), are reported to be increasingly demanded across borders ( Terzi *et al.* , 2008 ). However, these have hardly ever been studied in more detail. This study is the first to provide explorative insights into outbound medical travel for ophthalmological procedures. A quantitative content analysis of medical travel websites in German language was undertaken. Websites that support individuals in searching for and receiving these treatments abroad were identified via popular Internet search engines and the use of specific lay terms. The final sample consisted of 50 websites of providers and intermediate companies and were judged according to criteria such as information provided on clinicians, the clinic itself, offered treatments, pre- and post-care information, contact details and tourism-related services supplied. Each of these categories was subdivided into certain items, leading to 25 variables that were measured on a nominal “present/not present” scale (see Table 1 ). Furthermore, between October 2018 and March 2019, twelve semi-structured interviews were conducted with patients who reside in Austria and have gone abroad for refractive surgery. Interviewees were sought via multiple channels, including online invitations on social media platforms, information spread in the broad personal and work environment, a collection of patients' testimonials, and snowball sampling. The final sample consisted of patients aged between 23 and 40 years. Ten out of twelve interviewees were either fully or partially employed at the time of surgery and seven even had a tertiary degree.

With respect to the inbound flow, a similar approach was followed in a project examining the online efforts of Austrian hospitals in participating in medical travel by specifically targeting international patients through their web appearance ( Österle *et al.* , 2014 ). All hospitals that seemed to take part in medical travel, either through offering English translations of their websites and/or through being listed on a web portal dedicated to medical travel, were included in a final sample of 32 hospitals, varying in terms of size, range of medical specialties, and economic orientation (non-profit and for-profit). These websites again were analyzed according to their depth and attractiveness of information offered to foreign patients. Additionally, we draw on qualitative data gained from ten expert interviews (managers and medical doctors), of which three were conducted with representatives from Viennese for-profit hospitals, three with representatives from non-profit hospitals, one with a medical doctor active in both for- and non-profit hospitals and another three with employees from local and international medical travel agencies. The face-to-face interviews aimed at gathering information on the demographic and medical background of international patients treated in Austria and at getting expert insights into the complex interrelation of factors that determine the volume and the character of inbound medical travel. All interviewees, experts as well as patients, provided informed consent prior to data collection. Interviews were transcribed verbatim and a qualitative content analysis was undertaken to identify common themes ( Schreier, 2012 ).

Quantitative data on medical travel is largely limited on national and even more so on international level ( Lunt *et al.* , 2014a ). Limitations arise from a huge lack of systematic data collection in the private and in the public health care sector and from inconsistencies in (not) differentiating between different kinds of cross-border health care. That is why this study does not allow a comprehensive quantitative picture of inbound and outbound medical travel in Austria. However, the combination of earlier research and multiple methods helps to examine the different types of medical travel currently observable and to characterize the patient flows with regard to distinct procedures and drivers.

## 4. Medical travel to and from Austria

Across the literature, there is broad consensus on an increasing number of patients deliberately seeking health care abroad in the past decades, both on a global and on a European scale ( Chuang *et al.* , 2014 ; Lunt and Mannion, 2014 ). While availability and quality of data on medical travel is still scarce, earlier OECD data suggested that Central and Eastern European countries are major exporters in medical travel, supplying medical services to non-residents. In 2012, Croatia (5.29%), the Czech Republic (4.36%) and Hungary (4.25%) were the countries with the highest share of exports of health-related travel as percentage of their total health expenditure. Austria showed a share of 0.47% of total health expenditure and ranked among the countries with lower exports but is supposed to be an important source country of medical travelers ( OECD, 2014 ). These levels seem quite low compared to what people argue when asked about their willingness to travel abroad for health care. According to Eurobarometer surveys, about 40% of Austrian respondents are willing to go abroad for care, with even higher rates among younger age groups and those who already received a medical treatment in another EU member state within the past year. Among all interviewed EU citizens, almost 50% of respondents indicate a willingness to travel abroad for treatment ( European Commission, 2007 , 2015 ).

In the next two sections, inbound and outbound medical travel are explored for the Austrian case. The analysis particularly addresses the motives for travelling, the types of treatments demanded as well as the providers' orientation towards medical travel and other services offered to patients.

### 4.1 International patients in Austria

In contrast to many Eastern European and Asian countries successfully engaged in the medical travel market, Austria represents a rather highly priced destination country. While reliable data on the actual scope of inbound medical travel is missing, the country has a longstanding tradition in providing high-quality care to patients from abroad. The most prominent destination for planned treatment in Austria is Vienna; hence, the following exploration focuses on inpatient care of international patients in the Austrian capital city.

Findings from the expert interviews suggest that the majority of international patients in Viennese hospitals originate from Russia, from Eastern European countries outside the EU and Eastern European EU members states. Russian citizens, most of them residing in major cities such as Moscow, Saint Petersburg or Krasnodar, represent a comparatively large share of medical travelers in Vienna, followed by patients from Ukraine, Kazakhstan, Azerbaijan, Belarus, Georgia, Chechnya, Serbia and Turkey. Historically, Austria used to be attractive also for medical travelers from Arab countries. Their share, however, has steadily declined as a consequence of improvements of their own health systems (not least to develop these countries into medical travel destinations) but also due to new medical travel destinations for patients from Arab countries. From within the EU, most medical travelers in Viennese hospitals stem from Romania, to less extent from Hungary, Poland, Slovakia or Bulgaria. In total, the share of international patients in Viennese hospitals – defined as patients visiting Vienna for planned medical care – is low. Only few private hospitals report proportions of international patients' inpatient stays reaching 10% of the total number of patients. Unsurprisingly, the share of international patients is higher in hospitals that specifically promote their services to foreign patients on their websites, most importantly private hospitals.

With regard to the motives for care abroad, our findings indicate that failures of the domestic health systems, of specific hospitals or specialized departments are major reasons for international patients to seek care in Vienna. Especially in the case of Russia, Ukraine or Romania, the lack of inadequate medical infrastructure in both public and private hospitals seems to encourage patients to obtain care outside their home health care systems, provided that they can afford it. Mostly, medical travelers are paying treatments in Austria out-of-pocket. There are however also patients with more challenging economic backgrounds that have to mobilize multiple financial sources to receive necessary care. Pull factors for choosing Austria as medical travel destination are diverse. They include factors that are also applicable to other health care systems, such as the good reputation of hospitals, the high-quality care provided or privacy issues. Earlier visits to Vienna, social networks, the reputation of Vienna as a (medical) travel destination and the opportunities for easy travel also contribute to the choice of Viennese hospitals.

From the supply-side perspective, private hospitals in Austria openly value medical travelers as an additional income factor. However, different from some of the most prominent medical travel destinations, hospitals remain more cautious in terms of increased medical travel. Referring to historical and international experiences, they emphasize the risk of very substantial and possibly rapid fluctuations. Hence, even in the private sector, the primary focus is not on international medical travelers, but on domestic patients. Consequently, some private hospitals invest in efforts to facilitate medical travel, while others are more reluctant in actively targeting international patients. Hospitals with a public health care mandate and planned infrastructure to fulfil that mandate have a more ambivalent attitude towards patients from abroad. In general, treating foreign patients in these hospitals is not stimulated by financial reasons. They do not actively search for international patients. Rather, it is a combination of individual medical needs and an existing professional or social relationship. This could be between hospitals across countries, between doctors or even a personal relationship between doctors and prospective patients that initiate such treatments. In fact, according to the experts interviewed, informal networks, especially word-of-mouth recommendations, and the Internet as an increasingly popular tool for reaching international patients are the most influential factors for selecting a certain provider and destination country. This is also confirmed in the existing literature ( Hanefeld *et al.* , 2015 ). The underlying decision-making process however is complex and co-determined by various factors in the social, institutional and legal context ( Heung *et al.* , 2010 ; Runnels and Carrera, 2012 ).

Medical treatments typically demanded by international patients in Viennese hospitals are routine check-ups, oncological procedures, and orthopedic interventions. On a more general level, these procedures stand out because they allow for traveling. The availability of excellent medical professionals and modern medical techniques in Austria attract patients with specific needs. Apart from routine check-ups and second opinion services from Austrian specialists, oncology is a highly demanded specialty. Since oncological treatments often require multiple visits in frequent time intervals, the geographical distance between country of origin and country of treatment becomes a decisive factor. Vienna in this respect clearly benefits from its central location in Europe, easy to reach from Eastern Europe.

Hospitals regularly welcoming international patients also provide a range of support services, e.g. assistance for visa requests. Hospitals also indicate to be well prepared for treating non-German speaking patients. Most hospitals collaborate with a network of freelance translators who support physicians and nurses, if necessary. Nursing staff also often provides language skills that can help treating international patients adequately. In many cases, a translator is not needed by medical travelers, as they speak English or German themselves, or, in some cases, prefer to travel with their own translators. More extended incoming services, e.g. touristic activities, are seldom offered.

### 4.2 Austrian patients going abroad

There is broad consensus on both the historical and the current relevance of Austrians travelling abroad for medical treatments. The Austrian Health Interview Survey 2006/07 suggests that about 3% of the Austrian population, and 5% of Viennese citizens, received at least one medical treatment abroad in the past year, not differentiating between planned and unplanned treatment abroad. One-third of treatments refers to dental services ( Klimont *et al.* , 2007 , pp. 46-47). Cross-border travel for dental care has a longstanding tradition in Europe, with Hungary playing an outstanding role as destination country. For the Austrian case, it is the only example of planned cross-border health care that has been studied in some detail ( Kovacs and Szocska, 2013 ; Österle *et al.* , 2009 ).

While Hungary evidently is a main destination for dental tourists not just from Austria but also from other countries, we also sought to identify providers and intermediaries in Europe that promote refractive treatments (in particular procedures to correct myopia, e.g. LASIK) to German speaking patients. According to the systematic analysis of medical travel websites, most providers and intermediaries identified were placed in Turkey (33 out of 50 websites investigated); a country that has become a frequently advertised destination for ophthalmological services. With the political situation becoming more instable in Turkey, mainly Central Eastern European countries, in particular Poland, Slovakia, Hungary, and the Czech Republic, have become prominent destination countries for ophthalmological procedures. Generally, the medical travel market in the Central Eastern European region is said to be considerable given the economic, political and cultural ties between countries ( Horsfall, 2019 ).

In a demand side perspective, outbound flows of Austrian citizens residing in the Eastern part of the country are driven by geographical vicinity and comparatively lower costs. The fact that specific treatments are fully or partly excluded from public health insurance coverage encourages Austrians to seek more affordable alternatives across borders. While Austria and most other European countries are characterized by covering the (almost) entire population with an extensive health care package, elective treatments in a couple of fields require substantial co-payments or are even completely excluded from reimbursement via public health care systems. This applies to many dental treatments and ophthalmological procedures. Studies on dental care confirm the importance of costs as a major driver for medical travel ( Österle *et al.* , 2009 ; Kovacs and Szocska, 2013 ). If patients expect a similar level of quality in health care, the lower the payments for health care in another country compared to the home country, the higher a patient's incentive to go abroad ( Starmans *et al.* , 1997 ). In practice, however, the assessment of factual medical quality is difficult to assess by patients. Hence, it is rather perceived medical and service quality that matters for patients ( Han and Hyun, 2015 ).

The assumption that costs might be one decisive factor for outgoing Austrian patients in the field of dental care and ophthalmology, but also for other medical branches such as cosmetic surgery, is supported by results drawn from the analysis of interviews and of websites promoting these services to foreign patients. Most of them highlight the comparatively low costs for good quality treatments, in some online cases even arguing that a treatment combined with a holiday stay would cost prospective patients less than obtaining similar treatments at home. The findings also suggest that Austrians, similar to international patients in Viennese hospitals, to a large extent travel abroad for procedures they are paying out-of-pocket. Planned outbound medical travel based on cross-border health insurance schemes, by drawing on European social security coordination regulations, so far plays only a more marginal role.

From a supply side perspective, medical travel in the field of dental and ophthalmological care developed quite differently, indicating major differences in the providers' orientation towards medical travel. Dental tourism in Eastern Europe can be described as a bottom-up development, originally facilitated by small practices of dentists ( Österle *et al.* , 2009 ). In contrast, medical travel in the field of ophthalmology has evolved rather as a top-down strategy. In the Turkish case, similar to developments in Asian countries such as Thailand and Malaysia, the government has created the necessary policy framework and has heavily invested in the expansion of private health care provision with the intention to treat affluent patients from abroad ( Kaya *et al.* , 2015 ). Efforts have been made to engage interested stakeholders in the medical travel industry and to promote the country's service quality and competitiveness. This can be exemplified by co-operations of health care providers with the parastatal Turkish Airlines for attracting international patients, e.g. through special discounts and other incentives specifically made for medical travelers ( IMTJ, 2015 ). In Eastern European countries, it are more often international companies or medical doctors investing in private clinics. Taking advantage of lower infrastructure costs, they are aiming at more affluent patients from these countries and from abroad.

Apart from dental care and ophthalmology, we can conclude from our website analysis that a relatively wide range of medical treatments is advertised in German language through medical travel websites, also including assisted reproductive technologies, hair transplantation and cosmetic surgery. Different from other health provisions, many of these treatments are excluded from reimbursement via public health insurance. They are treated as electives and can be planned in advance, making them good candidates for medical travel ( Österle *et al.* , 2013 ). In the case of ophthalmological procedures, our study shows that almost all websites provide some treatment related information, even though the quality and amount of information regarding specific procedures varied considerably, ranging from precise explanations and credible descriptions to cursory glances on the various procedures offered. A lack of detailed and reliable medical information has also been identified by other exploratory studies ( Horsfall *et al.* , 2013 ; Lunt and Carrera, 2011 ). Given that most websites belonged to facilitators, they also contain tourism-related services to help medical travelers while travelling and staying abroad. This is another indication that outbound flows are strongly linked to tourism patterns. From a health policy perspective, this could become a problematic trend, potentially downplaying the risks of complications that can arise after being treated abroad, which then again can cause substantial costs in domestic public health systems. Findings from the interviews, however, show that while most patients appreciated accompanying services such as hotel booking and transportation service, an intermediary agency was contacted only in one single case. All other patients contacted providers directly and selected them based on personal recommendations, social networks, geographical vicinity and cultural links rather than sole industry marketing. Eight out of twelve interviewees went to Bratislava for refractive surgery, two to Kairo, and each one to Budapest and New Delhi.

### 4.3 Key findings and further considerations

While a lack of quantitative data still limits research in medical travel, in this study, the combination of findings from several projects allows to disentangle the patterns of inbound and outbound medical travel in Austria. As a health care destination, the country can benefit from its geographical position in Central Europe. International patients choose Austria largely for quality reasons, but in varying combinations with the perception of the broader economic and social situation, the reputation of providers, and accessibility in terms of travel or personal relationships. According to the expert interviews, international patients appreciate the Austrian mindset, cultural heritage, and landmarks. From a patient perspective, Austria is a comparatively highly priced destination, which limits the scope for inbound medical travel. Focusing on the supply side, evidence suggests that some inpatient providers actively pursue the acquisition of international patients, but none of the Viennese hospitals investigated relies on cross-border health care as their main income source. Different from countries in Asia and some European countries or regions, there is no economic policy strategy making medical travel a priority. Hence, the inbound flow of international patients remains a strongly patient-driven trend. Individuals are searching or considering health care in Austria to overcome the (assumed) lack of medical expertise and resources in their home countries. The majority of these international patients in Viennese hospitals is paying out-of-pocket. Applying the typology by Glinos *et al.* (2010) , inbound medical travel in Austria is predominantly characterized by patients travelling without cross-border insurance coverage given the motivational factors of availability in terms of specialized care and perceived high quality provided in Austria.

In contrast, private hospitals, specialized clinics, and doctors in Eastern European countries have established themselves as “key players” in the medical travel market with the aim to attract patients from abroad. These countries are emphasizing medical travel as a growth sector and driver of economic development, and, in some cases, also as facilitator for developing the local health care system. While an outbound flow of Austrian patients may lead to a limited financial burden for public health care budgets in the source country or a shortfall of revenues in the local private health care market, it may serve as growth engine in less advanced countries that can provide quality health care at lower costs. Austrians travel abroad for treatments that are easy to plan, elective rather than essential, and at least partially excluded from public health insurance. Limitations in public health care coverage and necessary out-of-pocket payments are decisive factors for outbound medical travel, as for dentistry or ophthalmology. Referring to Glinos *et al.* (2010) and Österle *et al.* (2013) , outbound patient flow is very much driven by economic concerns, and – in cases such as assisted reproductive technologies – availability. A major co-determinant is service quality, very much promoted by facilitators and providers.

To sum up, outbound medical travel is largely determined by factors of access, affordability and vicinity, while inbound medical travel is predominately determined by quality, both in terms of medical quality and broader service quality, and a lack of adequate medical infrastructure in the source countries. Despite these commonalities, the development and dynamics of inbound and outbound medical travel are highly dependent on social and cultural factors and vary largely for specific procedures. Overall, however, developments are characterized by patients acting as consumers of care and by a growing supply addressing and facilitating cross-border travel. Besides that, patient flows in Europe are also taking place outside the competitive medical travel market. The EU 2011 directive on planned cross-border health care in Europe, allowing for reimbursement of planned treatments abroad if they fall under the benefit package of the competent state, may facilitate these developments further. Existing frameworks studying planned medical travel abroad have largely focused on the demand side perspective (and the motives for travelling abroad) as well as the legal context for reimbursement, while coverage of the supply-side remains more limited. Hence, in the next section, this paper argues for a more policy-driven approach based on empirical knowledge and proposes an extended general framework that can guide future research on analyzing both demand and supply of international medical travel.

## 5. Discussion: towards an extended framework for analysis

As the Austrian example confirms, medical travel is a multifaceted phenomenon. While patient-driven movements across borders for health care are not new, social and economic changes of the past decades have clearly accelerated the transnational demand for and supply of health care across borders. Changes towards a more individualized health culture ( Parr, 2002 ) conceptualize patients as their own health experts ( Ormond and Sothern, 2012 ). Medical travel incorporates this development. It are increasingly patients themselves who decide where to go and what kind of treatment to receive abroad, often reflected by social ties rather than industry marketing. Their choices strongly depend on individual circumstances and social networks. While most research focuses on patients' motives to seek care in another country (demand), this study incorporates the providers' perspective towards medical travel (supply) and the role of intermediaries.

**Figure 1:**
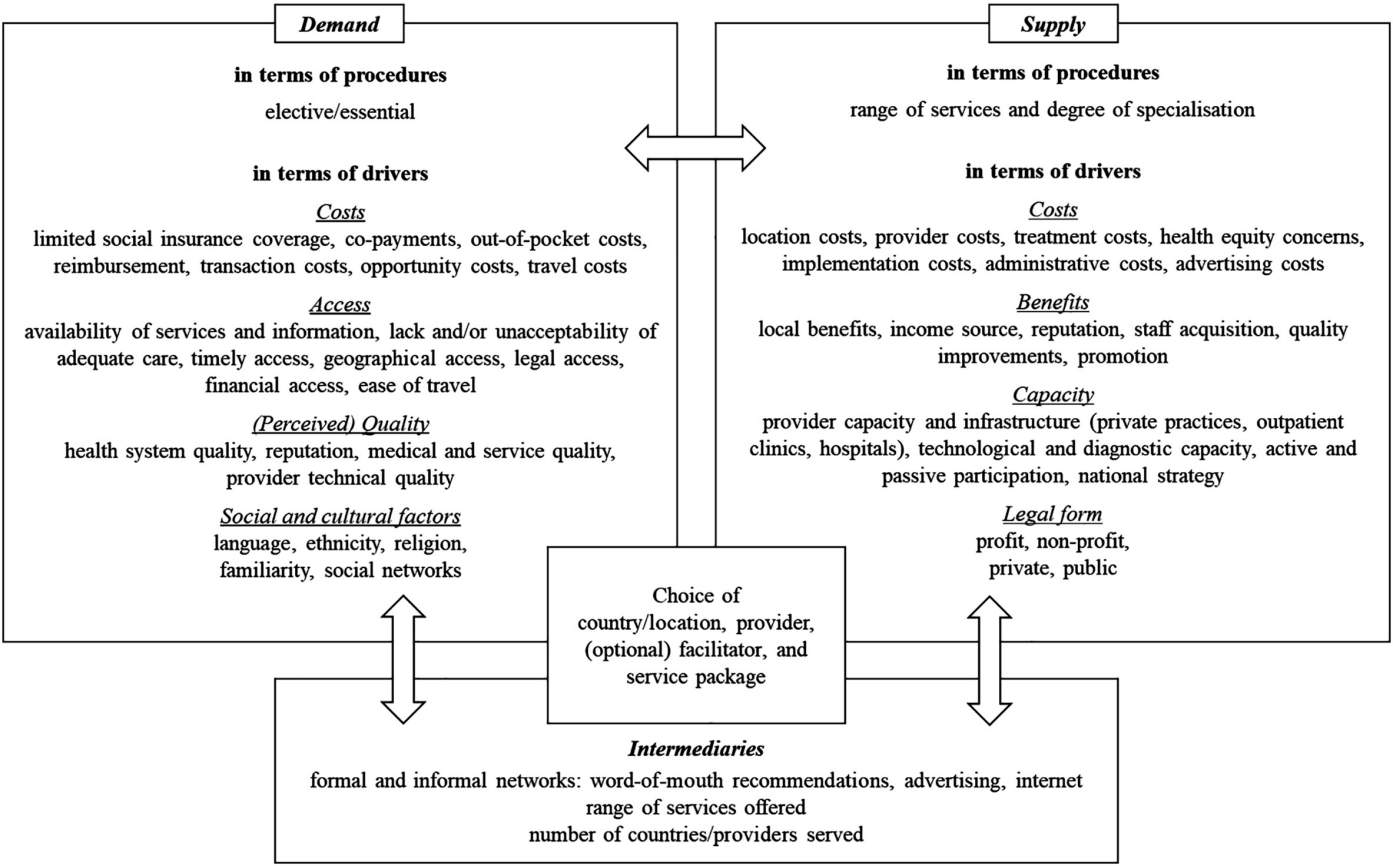
Conceptual framework on the demand and supply of medical travel

Bringing both demand and supply-side determinants together (see Figure 1 ), the study offers an extended conceptual framework applicable for diverse patient flows. Similar to earlier studies, on the demand side, the framework differentiates between elective and essential procedures and identifies a range of drivers. These are clustered under the headings of costs, access, (perceived) quality as well as social and cultural factors ( Glinos *et al.* , 2010 ; Österle *et al.* , 2013 ).

On the supply side, in terms of procedures, a wide range of services and different degrees of specialization are possible. In general, the possibility to assess the options in advance is a requirement for these procedures. In terms of the supply side drivers for medical travel, these are clustered as costs, benefits, capacity and legal form. Regarding costs, beyond those that also incur for local patients, costs for ensuring and marketing high quality medical care, the upgrading of non-medical services, advertising, or services for international patients such as welcome services or translation services, need to be considered. In terms of benefits, apart from an additional income source, the reputation as a provider for international patients or becoming more attractive for staff acquisition are potential drivers of engaging in medical travel. If the infrastructure is not established for the initial purpose of serving international patients, capacity and infrastructure – at least in a medium-term perspective – can become a limiting factor. This refers to technological infrastructure, but also to capacities in terms of personnel. In addition, strategies promoting growth in medical travel – be it by countries, regions or networks of providers – can further help establish a region as a medical travel destination. Partly related to that, the institutional context and legal forms matter. Public hospitals and publicly funded hospitals often have a public mandate limiting their opportunities to engage in planned medical travel.

Besides demand and supply, intermediaries play an important role in facilitating medical travel. These can take very different forms. In the early developments, it are usually users or providers establishing medical travel, it are patients choosing doctors or clinics abroad for treatments, or doctors and clinics addressing potential patients abroad. In the process of developing and expanding medical travel, facilitators become more important actors. They could be former patients, providers that start to act as facilitators or traditional intermediaries moving into this specific market segment. Brokerage and intermediation are increasingly organized via Internet platforms, but more personal intermediation by individuals or (medical) tourism agencies are still common. They specialize on specific treatments, on specific countries or regions, or they provide a range of procedures and destinations.

So far, analyzing the supply and brokerage of medical travel has primarily been left to marketing and tourism studies ( Heung *et al.* , 2010 ), whereas research from a policy perspective that deals with both demand and supply of medical travel remains relatively scarce. With its emphasis on both individual and systemic supply and demand-side factors, Figure 1 outlines the main empirically identified characteristics in which actors can be differentiated that shape inbound and outbound patient flows and affect health organization and management. By proposing this framework, we seek to address this gap and help to disentangle patient flows moving beyond just distinguishing a few dominant medical travel patterns as “old” and “new” and by acknowledging that these and other forms of medical travel in Europe coexist.

Undoubtedly, an approach including both inbound and outbound medical travel broadens the research focus and might limit the possible depth of analysis. However, we argue that it allows for a stronger synopsis of different factors determining patient flows in various directions and for a variety of treatments. Taking different circumstances into account finally also helps to derive an extended framework that is suitable for more policy-centered analysis considering not only patients' demand but also health care supply to foreigners, which can be far more complex than hitherto discussed. While early contributions have definitely dealt with medical travel induced policy challenges such as equity concerns in destination countries ( Johnston *et al.* , 2010 ), the phenomenon at its very basic definition comprises much more than the North-South divide in transnational health care ( Virani *et al.* , 2020 ). Policy deficiencies exist in typical source countries, such as extensive waiting times, as well as in destination countries all over the globe, and can often be directly related to patients' motivations or providers' orientation towards medical travel. This interplay still needs to be addressed in more detail, globally and at European scale, and against the specific country and health system context. Going beyond single procedure studies, improving availability and comparability of hard data on cross-border health care, more closely linking demand and supply perspectives and using evidence from different settings will help develop more empirically-grounded policy advice.

## 6. Conclusion

This study provides empirical evidence on medical travel to and from Austria. Analyzing both inbound and outbound medical travel allows to assess patient flows for different drivers and procedures relevant for contemporary medical travel. Apart from the dominant industry and tourism perspective that is based on the approach of “consumers in search of value” ( Deloitte, 2008 ), medical travel still takes place in a wider context driven by socioeconomic, political, cultural, and familiar ties between countries. This is reflected by the drivers proposed in the framework, applicable for diverse forms of medical travel. While demand for medical travel is driven by affordability, accessibility, quality and familiarity, health care supply to international patients is common but not limited to private market providers. It varies largely in terms of costs, benefits, capacity and legal form of providers. By using the example of Austria and suggesting an extended framework for medical travel, this study argues for a stronger policy-sided approach and a more nuanced acknowledgment of diverse medical travel patterns existing in Europe.

## Abbreviations

EEA –European Economic AreaEU –European UnionLASIK –Laser-in-situ-KeratomileusisOECD –Organization for Economic Co-operation and DevelopmentNHS –National Health Service, the publicly-funded health care system of the United Kingdom

## Figures and Tables

**Table 1:** Content criteria for the systematic review of medical travel websites

Category/Item	(1)	(2)	(3)	(4)	(5)
(1) Clinicians	Name	Experience/qualification	Language skills	Photo	
(2) Clinic	Location (country, city)	Accreditation	Number of professionals	Number of surgeries	
(3) Offered treatments	Ophthalmological treatments, *e.g. refractive surgery, cataract surgery, etc.*	Other types of treatments, *e.g. dental care, cosmetic surgery, etc.*	Treatment related information	Estimated costs	
(4) Pre- and post-care	Information about pre-care	Information about follow-up care	Procedural risks	Existing complaints procedure	
(5) Contact	E-mail	Telephone	Information request/contact form	Testimonials/patients feedback	
(6) Tourism-related services	Air travel	Accommodation	Translation services	Transportation services	Vacation option
